# Ubiquitin and Parkinson's disease through the looking glass of genetics

**DOI:** 10.1042/BCJ20160498

**Published:** 2017-04-13

**Authors:** Helen Walden, Miratul M.K. Muqit

**Affiliations:** 1 MRC Protein Phosphorylation and Ubiquitylation Unit, School of Life Sciences, University of Dundee, Dundee, U.K.; 2 School of Medicine, Dentistry & Nursing, University of Dundee, Dundee, U.K.

**Keywords:** kinase, Parkinson's disease, ubiquitin, ubiquitin ligases

## Abstract

Biochemical alterations found in the brains of Parkinson's disease (PD) patients indicate that cellular stress is a major driver of dopaminergic neuronal loss. Oxidative stress, mitochondrial dysfunction, and ER stress lead to impairment of the homeostatic regulation of protein quality control pathways with a consequent increase in protein misfolding and aggregation and failure of the protein degradation machinery. Ubiquitin signalling plays a central role in protein quality control; however, prior to genetic advances, the detailed mechanisms of how impairment in the ubiquitin system was linked to PD remained mysterious. The discovery of mutations in the α-synuclein gene, which encodes the main protein misfolded in PD aggregates, together with mutations in genes encoding ubiquitin regulatory molecules, including PTEN-induced kinase 1 (PINK1), Parkin, and FBX07, has provided an opportunity to dissect out the molecular basis of ubiquitin signalling disruption in PD, and this knowledge will be critical for developing novel therapeutic strategies in PD that target the ubiquitin system.

## Introduction

Approximately a century ago, Friedrich Lewy made a key discovery towards the understanding of Parkinson's disease (PD) by identifying and describing large cytoplasmic proteinacious inclusions in brains of patients who had died with the disease [1]. These inclusions, subsequently termed Lewy bodies, were distinct both in their morphology and location from inclusions found in other neurodegenerative diseases, e.g. extracellular amyloid plaques of Alzheimer's and intranuclear inclusions of Huntington's disease [2]. Furthermore, their preferential location in those regions most affected in Parkinson's including within surviving dopaminergic neurons of the pars compacta firmly established Lewy bodies as one of the key neural substrates of PD. Whether Lewy bodies are harmful to neurons or a protective response remains controversial. However, seminal pathological studies in the 1980s found that the majority contained a small protein, ubiquitin [3], which had been discovered in the preceding decade [4] and shown to be a critical modifier that tagged proteins for degradation [5]. Therefore, the identification of ubiquitin in Lewy bodies provided strong evidence for the role of altered ubiquitin signalling and disrupted protein quality control in PD. However, the molecular insights into how ubiquitin controlled these processes including the key enzymes involved in mediating ubiquitylation, the identification of key ubiquitylated substrates that reside in inclusions, and the key components controlling the reverse pathway remained unknown.

## The ubiquitin system and Parkinson's genetics

Advances in genetics have begun to unravel the molecular basis of Parkinson's through the discovery of nearly 20 genes mutated in rare familial forms of the disease. The proteins encoding these genes have been implicated in diverse cellular pathways including mitochondrial quality control [Parkin, PTEN-induced kinase 1 (PINK1), and DJ-1]; protein misfolding, and aggregation (α-synuclein); membrane trafficking and autophagy (α-synuclein, LRRK2, PINK1, and Parkin); and synaptic function and vesicle release (α-synuclein, Synaptojanin, and TMEM230) [6]. Strikingly from a molecular standpoint, several of these genes encode direct components of ubiquitin signalling (Parkin and F-box only protein 7 (Fbxo7)), regulators of ubiquitin signalling (PINK1), or key target substrates of ubiquitin signalling (α-synuclein). This has provided a framework to explore how mutations in these genes have an impact on ubiquitylation, revealing a crucial role for ubiquitylation in regulating protein quality control pathways whose stress-induced dysregulation underlies Parkinson's linked neurodegeneration.

**Figure 1.**
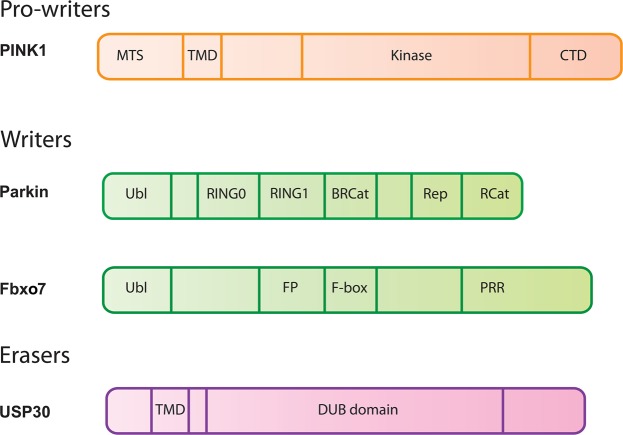
Domain schematic of key ubiquitin-related proteins in PD. Ubiquitin molecules that are either mutated in PD (PINK1, Parkin, and Fbxo7) or are a leading candidate for drug discovery (USP30) are depicted. PINK1: MTS, mitochondrial-targeting sequence; TMD, transmembrane domain; CTD, C-terminal domain. Parkin: Ubl, ubiquitin-like domain; RCat, required for catalysis; BRCat, Benign Rcat; Rep, Repressor element of Parkin. F-box07 (Fbx07): Ubl, ubiquitin-like domain; FP, Fbxo7 and PI31-interacting domain; PRR, proline-rich region. Ubiquitin-specific processing protease 30 (USP30): TMD, transmembrane domain; DUB, deubiquitinase domain.

Ubiquitin, an 8.5 kDa polypeptide, was first isolated from bovine thymus and subsequently found to be expressed in diverse tissues of mammalian cells, yeast, bacteria, and plants, but for several years its function was unknown [4]. Borne out of motivation to better understand the nature of protein degradation, pioneering studies elucidated a multistep ATP-dependent enzymatic cascade by which ubiquitin is conjugated to proteins, marking them for degradation [7]. The action of three enzymes, namely an Ub-activating enzyme (E1), Ub-conjugating enzyme (E2), and the Ub-ligating enzyme (E3), catalyses covalent attachment of Ub to a substrate via an isopeptide bond between the ε-amino group of the substrate lysine and the C-terminal Gly residue of Ub [8]. In addition to the target lysine of substrates, ubiquitin also possesses seven internal lysine residues [Lys6 (K6), Lys11 (K11), Lys27 (K27), Lys29 (K29), Lys33 (K33), Lys48 (K48), and Lys63 (K63)] and an N-terminal amino group that add complexity via generation of homotypic and heterotypic chain linkage types. These bring diversity to the downstream signalling of distinct chain types: for example K11 and K48 chains tag proteins for degradation via a large protease complex, the 26S proteasome. In contrast, K63 chains confer non-degradative effects particularly in recruiting proteins to sites of DNA damage or following activation of Toll-like receptors in the innate immune response [8]. The enzymes that catalyse ubiquitylation are often termed *writers* of the ubiquitin system and include two PD-linked E3 ligases, Parkin and Fbxo7 (Figure 1). The major PD-linked ubiquitylated *target* to date has been α-synuclein, and the identification of α-synuclein as the major protein component of Lewy bodies has led to extensive research to address the regulation of α-synuclein misfolding by ubiquitin and how this influences its degradation and downstream signalling in neurons [1]. In parallel, the cell contains a host of adaptor proteins that possess domains capable of recognising specific Ub-chain types. Approximately 20 ubiquitin-binding domains (UBDs) are known and are present in several hundred proteins. Such *readers* are capable of decoding the ubiquitin signals and inducing concomitant signalling events [9]. Of relevance to Parkinson's are several UBD adaptors that are critical to the 26S proteasome-mediated recognition and degradation of oligomerised α-synuclein, and recently, Optineurin and NDP52 have been identified to signal downstream of Parkin-catalysed ubiquitylation at mitochondria. Finally, deubiquitinating enzymes (DUBs) cleave Ub from substrates or chains, thereby enabling recycling of ubiquitin. These *erasers* include six subfamilies including the ubiquitin carboxy-terminal hydrolases (UCHs), which is of relevance since mutations in one member UCH-L1 were reported in 2000 in a pair of siblings with Parkinson's. However, whilst there is much known on the biological function of UCH-L1, its genetic link to Parkinson's remains to be confirmed. There is growing interest in the role of ubiquitin-specific proteases (USPs) in their regulation of mitochondrial quality control pathways linked to Parkinson's (USP30 and USP15) and in the cleavage of ubiquitin chains conjugated to α-synuclein (USP8). There are as yet no links of the other family members of DUBs including Machado–Joseph disease protein domain proteases, ovarian tumour (otubain) proteases (OTU), JAB1/MPN/Mov34 metalloenzyme motif proteases (JAMM), and the newly discovered motif interacting with Ub-containing novel DUB family) (MINDY) proteases to Parkinson's pathways [10].[Fig BCJ-2016-0498CF2]

**Figure 2. BCJ-2016-0498CF2:**
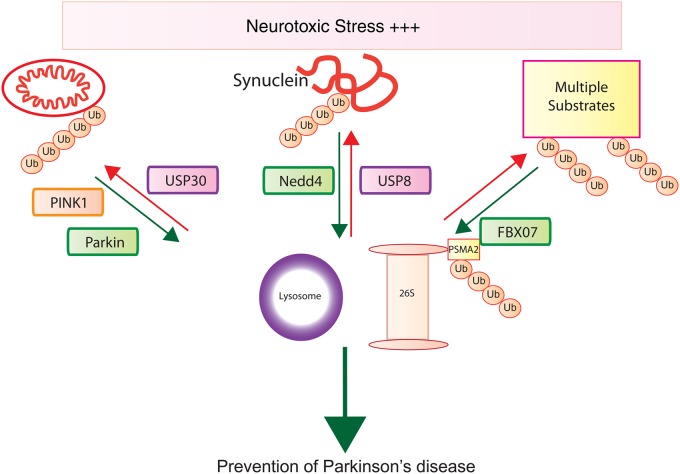
Ubiquitin pathways combating Parkinsonism. Multiple proteins and pathways implicated in protein homeostasis are shown. Ub, ubiquitin; USPs, ubiquitin-specific proteases; E3 ligases are shown in green and USPs in purple.

In addition to these enzymatic ‘erasers’ of ubiquitin signalling, a major consequence of ubiquitylation of proteins is degradation via the 26S proteasome, a multisubunit protease complex. K48-linked chains were initially found to signal proteins for 26S proteasome-mediated degradation but since then other linkage types can be recognised by the proteasome including K11 that can trigger degradation of various components of the cell cycle during cell division. To date, none of the genes encoding the 28 subunits of the proteasome are mutated in Parkinson's familial cases. Nevertheless, analysis of human postmortem brains of sporadic Parkinson's patients has suggested that proteasomal activity is impaired. In particular, it has been reported that proteasomal subunits and proteasomal activity are reduced in the substantia nigra but not in other brain regions of patients with PD [11]. It was further reported that chronic systemic administration of a proteasome inhibitor in rats led to a Parkinsonian phenotype associated with dopaminergic cell loss and importantly ubiquitin and α-synuclein-positive inclusions [12]. However, this model was not independently replicated by different laboratories and thus has not become an established model in the Parkinson's field. In contrast mitochondrial toxin-based models such as MPTP (1-methyl-4-phenyl-1,2,3,6-tetrahydropyridine) are widely used [13]. To address the role of the 26S proteasome further, a conditional knockout of the proteasome subunit Rpt2/PSMC1, encoding an ATPase of the 19S regulatory complex, has been generated in which this essential subunit was inactivated in the mouse substantia nigra. This led to extensive neurodegeneration of the nigrostriatal pathway and ubiquitin-positive inclusions; however, this was associated with death at 1 month, preventing assessment of whether there is a typical motor phenotype in this model [14]. Moreover, the degeneration and inclusions were found to occur independent of α-synuclein in follow-up work, suggesting that the mechanism of neurodegeneration in this model is not relevant to Parkinson's and indicates that the role of the ubiquitin system in preventing PD may be more complex in this model [14]. Finally, human brain studies cannot distinguish whether the proteasomal dysfunction seen in Parkinson's is a primary defect or a secondary consequence. Interestingly, a causal role for proteasomal dysfunction is emerging more widely from other neurodegenerative disorders through the identification of X-linked mutations in Ubiquilin2 (UBQLN2) in amyotrophic lateral sclerosis and the demonstration that UBQLN2 plays a crucial role as a proteasome shuttle factor clearing aggregates via the proteasome [15].

## α-Synuclein — ubiquitin and protein aggregation and turnover

The discovery of mutations in the α-synuclein gene in families with autosomal-dominant inherited PD represented a major advance in our understanding of PD, particularly with the demonstration that α-synuclein comprises the major component of Lewy body aggregates found within PD brains [1]. Overexpression of α-synuclein through gene duplications and triplications and disease-associated missense mutations stimulates the propensity of α-synuclein to aggregate and form fibrils *in vitro* and *in vivo* [1]. The regulation of α-synuclein-mediated fibril formation by post-translational modifications has been the subject of intense interest [16]. In particular, phosphorylation at Tyr39 and Ser129 has been shown to promote α-synuclein aggregation [17,18]. However, the role of ubiquitylation has been more controversial. Early studies focused on the role of ubiquitylation in the α-synuclein aggregation process and suggested that the seven in absentia homolog (SIAH) E3 ligase could target α-synuclein via ubiquitin multi-monoubiquitylation at Lysines 12, 21, and 23 and that the monoubiquitylated form of α-synuclein was more prone to aggregation both *in vitro* and *in vivo* [19]. However, the contribution of ubiquitin to promoting α-synuclein fibrillisation has been questioned since only a minor fraction of α-synuclein (∼10%) is ubiquitylated within Lewy bodies. Consequently, the role of ubiquitylation in regulating the physiological turnover of α-synuclein has become the focus of recent work. Several E3 ligases, including CHIP and E6-AP, have been suggested to ubiquitylate α-synuclein and mediate its degradation via the proteasome [20,21]. Whilst both CHIP and E6-AP have been reported to localise in the Lewy bodies, the mechanism by which they mediate α-synuclein degradation remains unclear, with data obtained from largely overexpression studies and the critical Lysine residues and ubiquitin chain topologies mediating degradation uncharacterised. K63-mediated ubiquitylation of α-synuclein (major sites of ubiquitylation at residues Lys21 and Lys96) via the Nedd4 HECT E3 ligase has been reported to signal for α-synuclein degradation via the lysosomal pathway (Figure 2) [22]. Furthermore, K63-linked ubiquitin was reported to be more abundant in Lewy body inclusions than K48 using Ub-chain-specific antibodies, although the authors did not specifically assay α-synuclein ubiquitylation [23]. Very little is known on the deubiquitinases (DUBs) that target α-synuclein, and a recent study has suggested that USP8 preferentially cleaves K63-ub chains attached to α-synuclein *in vitro* and knockdown of USP8 in cells accelerated α-synuclein degradation via lysosomes. Furthermore, USP8 knockdown prevented α-synuclein toxicity in a *Drosophila* model *in vivo* [23].

Little is known on the regulation of ubiquitin of the normal function of α-synuclein, which resides mainly in the synapses of neurons, where it has been postulated to control vesicle fusion in presynaptic terminals [1]. α-synuclein has also been reported to associate with other membrane compartments including the ER, Golgi, and endosomes to mediate membrane-lysosomal trafficking [1]. Previous studies in postmortem normal brains suggested that α-synuclein is not present as a ubiquitylated form; however, such analyses are susceptible to postmortem artefacts via the action of DUBs and proteases. Analysis of rat brain extracts with antibodies that recognise the Lys-ε-Gly-Gly (di-Gly) remnant of ubiquitylated proteins has suggested that residues Lys34 and Lys96 of α-synuclein are ubiquitylated [24]. Both these sites are conserved in the human protein and, interestingly, Nedd4 can ubiquitylate Lys96 of human α-synuclein *in vitro* [22]. Propagation of misfolded α-synuclein between neurons via exocytosis pathways, exosome production, and vesicle-mediated endocytosis is an area of intense interest; however, how ubiquitin controls these processes remains largely mysterious. Recently, the DUB, USP19, was shown to promote misfolded α-synuclein secretion [25]. USP19 possesses intrinsic chaperone activity that first recruits α-synuclein to the surface of the endoplasmic reticulum, where it is then deubiquitylated, encapsulated by late endosomes and finally secreted to the cell exterior [25].

## Parkin — autoinhibited E3 ligase and mechanism of activation

Mutations in the *PARK2* gene were discovered in 1998 in patients with autosomal-recessive juvenile Parkinsonism (AR-JP). They represent the leading genetic cause of early-onset Parkinson's (onset <45 years), accounting for 90% of all cases presenting before age 21 and ∼50% of those presenting before age 45. The patient phenotype is distinct with strong association with dystonia, slow disease course, and l-Dopa sensitivity both in terms of responsiveness and onset of dyskinesias. Pathologically, there is a striking absence of Lewy bodies in the majority of reported postmortem brain studies and relatively restricted pathology to the striato-nigral system unlike the more diffuse progression of pathology seen in sporadic cases. Interestingly, nearly all cases reported with Lewy bodies bear compound heterozygous Parkin mutations, and it is not clear whether Parkin activity is biochemically affected in a similar manner to patients harbouring homozygous mutations.

Parkin is an E3 ubiquitin ligase that is a member of the RING-Between-RING (RBR) family. There are ∼12 members of the RBR family in the eukaryal kingdom, and all feature a RING domain similar to those found in the RING family required for recruiting E2-conjugating enzymes (for recent reviews see [26,27]). In addition to the RING domain, RBRs have a catalytic cysteine that forms a catalytic intermediate [28], and a domain separating the RING and the catalytic domain, termed the InBetweenRING, or Benign-Rcat ‘B’ (Figure 1). All RBRs have additional domains to the RBR module, with Parkin having an N-terminal ubiquitin-like domain, which shares 30% sequence identity with ubiquitin, and a RING0 domain which is a linear zinc-binding domain [29]. Parkin is a 52 kDa protein comprising 465 amino acids [30]. Importantly, at least 80 pathogenic amino acid substitutions that lead to AR-PD are found throughout the primary sequence of Parkin, clustering in domains, but also in the linkers between domains [31]. The RBRs are usually autoinhibited via intramolecular domain–domain interactions and require activation [32–41]. In the case of Parkin, activation is achieved via two phosphorylation signals catalysed by PINK1. Serine65 (Ser65) of both the Ubl domain of parkin [42] and ubiquitin itself are phosphorylated by PINK1 [43–45]. Phosphorylated Parkin binds tightly to phosphorylated ubiquitin [34,46], and this triggers a conformational rearrangement that allows Parkin ubiquitin ligase activity [34,36,47]. The pathogenic mutations found in Parkin are not only interspersed throughout the primary sequence, but also throughout the structure of Parkin. Many mutations result in destabilisation of the various domains [35,40,48], leading to loss of ubiquitin ligase activity. Some mutations lead to inappropriate activation of Parkin, causing Parkin self-ubiquitylation and subsequent turnover by the proteasome [32,49]. Other mutants disrupt the ability of Parkin to perform the transthiolation necessary for activity [50]. In the 17 years, since the identification of Parkin as a ubiquitin ligase [51,52], many potential substrates of Parkin have been identified and reviewed [53–57]. Since ubiquitylation often acts as a signal for proteasomal degradation, there was an early expectation that Parkin substrates would accumulate in the absence of functional Parkin. However, in the multiple animal models of Parkin deficiency that have been generated, very few show increased levels of putative Parkin substrates (for a comprehensive review of mouse models, see ref. [58]). One notable exception is the accumulation of the aminoacyl-tRNA synthetase cofactor, AIMP2, which accumulates in brain tissue of Parkin–PD patients [59]. However, there is yet to be a comprehensive analysis of the levels of Parkin substrates in confirmed Parkin–PD cases.

Many of the candidate substrates of Parkin activity are found at the mitochondrial outer membrane and are involved in the maintenance of mitochondrial homeostasis. Indeed, our current understanding of both PINK1 and Parkin function is to drive the clearance of damaged mitochondria via mitophagy (recently reviewed in ref. [60]). PINK1 activity is required for recruitment of Parkin to damaged mitochondria [61–66]. Recent studies support a model whereby phosphorylation of ubiquitin, catalysed by PINK1 (Figure 2), is the signal for autophagy at the mitochondria, and that Parkin serves to amplify this signal [67].

## PINK1 — (phospho) ubiquitin, Parkin, and mitochondrial turnover

Mutations in PINK1 were identified initially in Sicilian patients with AR-PD [68]. Clinically patients exhibit a phenotype similar to Parkin patients with early age of onset, prominent dystonia, and sensitivity to L-Dopa. To date, three postmortem case studies have been reported with two noting the absence of nigral Lewy body pathology (homozygous C388R [69] and homozygous L347P [70]) and one finding Lewy body pathology (compound heterozygous for exon 7 deletion/5′ splice site mutation in exon 7 [71]). PINK1 encodes a 581-amino acid Ser/Thr protein kinase that possesses an N-terminal mitochondrial-targeting sequence and a kinase domain that is unusual due to the presence of several loop insertions and a C-terminal domain with no known homology to any other protein. Nearly 25 homozygous or compound heterozygous mutations have been reported for PINK1 with the vast majority lying within the kinase domain and affecting key residues critical for kinase function, e.g. the A217D mutation that occurs within the ATP-co-ordinating LAIK motif and causing childhood onset of PD [72]. Genetic and biochemical studies suggest that the majority of mutations are loss of function including truncating mutations that abrogate the C-terminus. Recently, a heterozygous mutation, G411S, was proposed as a risk factor for PD and acts in a dominant negative fashion as suggested from structural modelling by hampering putative PINK1 dimerisation [73]. Under basal conditions, PINK1 undergoes proteolysis by the PARL protease anchored to the inner membrane by Stomatin-like protein 2 (SPL2), which together with the protease YMEL1 forms the recently described SPY complex [74]. Cleavage of PINK1 generates a C-terminal fragment starting at residue Phe104 that signals for its degradation via the N-end rule pathway [75]. Upon mitochondrial depolarisation that can be induced by uncoupling agents [e.g. carbonyl cyanide *m*-chlorophenyl hydrazine (CCCP)], PINK1 becomes stabilised and activated and phosphorylates ubiquitin at the outer mitochondrial membrane via residue Ser65 [43–45]. The generation of Phospho-ubiquitin (p-Ub) stimulates recruitment of the Parkin, whereupon binding to p-Ub, it becomes efficiently phosphorylated at its N-terminal Ubl domain at Ser65 (equivalent to the Ubiquitin site) to become fully activated via a feed-forward mechanism. The consequent ubiquitylation of substrates by Parkin creates a feedback amplification loop whereby ubiquitin chains are further phosphorylated by PINK1 to boost the p-Ub at the mitochondrial surface (estimated to represent ∼20% of total mitochondrial ubiquitin), and this stimulates further Parkin recruitment and activation [46,76,77]. The mechanism of PINK1 activation is attributed to protein stabilisation and dimerisation and additional modifications may play a role including phosphorylation [78,79]. PINK1 resides in an ∼700 kDa complex associated with translocase of outer membrane members (TOM) that appear critical for its import [80]. Recent studies highlight a role for p-Ub in the recruitment of ubiquitin adaptors including Optineurin for induction of mitophagy via activation and phosphorylation of TBK1 although the mechanism by which TBK1 is activated and the identity of its upstream kinase are unknown [67,81]. Intriguingly, Optineurin is a recognised effector of the Rab8A GTPase [82], and phosphoproteomic analysis has revealed that Rab8A and related Rab GTPases 8B and 13 are phosphorylated at residue Ser111 indirectly in response to PINK1 activation via an unknown intermediate kinase [83]. In future work, it would be interesting to assess whether TBK1 can directly phosphorylate Rabs or, conversely, whether Rabs are required for optimal TBK1-mediated phosphorylation of Optineurin. There are probably additional downstream functions of p-Ub and studies *in vitro* suggest that p-Ub can influence interactions with DUBs [84]. Whilst the phosphatase that dephosphorylates p-Ub remains unknown, two DUBs have been identified that deubiquitylate Parkin-directed substrates, USP30 and USP15, and USP8 has also been reported to reverse Parkin autoubiquitylation.

Upon mitochondrial depolarisation, the ultimate fate of PINK1 and Parkin activation is degradation of mitochondria via mitophagy. In recent years, other forms of mitochondrial quality control have emerged including PINK1-dependent regulation of mitochondrial-derived vesicles (MDVs) that are cargo-specific in response to mitochondrial damage [85]. The MDV pathway appears to employ distinct downstream machinery since the SNARE protein, syntaxin-17, is required for delivery of MDVs to the late endosome–lysosome but is not required for mitolysosome formation and mitophagy [86]. MDVs have also been linked to physiological regulation of antigen presentation [87]. To date, it remains unknown whether perturbation of PINK1 and Parkin leads to defects in mitochondrial quality control in Parkinson's derived cells or tissues. However, the development of state-of-the-art cellular models and *in vivo* reporters will soon enable this question to be addressed. Recently, advances in cellular reprograming have led to the development of iPS-derived midbrain dopamine neurones from Parkinson's patients harbouring PINK1 and Parkin mutations, and these cells demonstrate aberrant α-synuclein cytosolic accumulation and mitochondrial defects, making them an ideal *in vitro* system to probe the role of mitophagy in PD [88]. Furthermore, the development of two *in vivo* mouse reporters of mitophagy will aid in validating the role of mitophagy in relevant PD mouse models [89,90].

## Fbxo7 — ubiquitin and new frontiers

A second member of the E3 ubiquitin ligases, Fbxo7, is also mutated in early-onset Parkisonism [91–97]. In contrast with Parkin, which functions as a single-polypeptide E3 ligase, Fbxo7 is a component of a multisubunit E3 ligase of the Cullin-RING ligase family (CRL). CRLs are a large class of multisubunit E3 ubiquitin ligases that feature a scaffolding protein, termed a cullin, and associate with a RING-box protein (reviewed in refs [98,99]). The archetypal CRL is the SCF-type ligase family, which comprises Cullin1, Skp1, the RING protein Rbx1, and an F-box. Skp1 associates with substrate adaptor proteins that contain F-boxes (Skp1–Cullin1–Fbox), which recruits substrates to the CRL for ubiquitylation.

Until recently, Fbxo7 had only four identified substrates. The first of these is HURP (hepatoma up-regulated protein) [100]. HURP is found at high levels in hepatocellular carcinomas and is required during mitotic spindle assembly for correct alignment of chromosomes [101]. As such, it is subject to turnover and is regulated by both Fbxo7 [102] and the anaphase-promoting complex [103]. Additional substrates include the cellular inhibitor of apoptosis (cIAP1) [104] and TNF-α receptor-associated factor 2 (TRAF2) [105]. cIAP1 and TRAF2 are both components of the NF-κB signalling pathway, which is the main driver of the inflammatory response. Interestingly, both of these substrates are E3 ligases themselves, a keen example of the role ubiquitylation plays in the regulation of NF-κB signalling (reviewed in ref. [106]). Fbxo7-mediated ubiquitylation of each of these prevents their association with the receptor-interacting protein 1 and inhibits NF-κB signalling [105]. However, as well as exerting an inhibitory effect on NF-κB signalling, Fbxo7 also targets a fourth substrate for ubiquitylation, Neurotrophin receptor-interacting homologue (NRAGE) [107]. NRAGE is also a component of the NF-κB signalling pathway, but in contrast with the effects via cIAP1 and TRAF2 ubiquitylation, modification of NRAGE leads to the formation of NRAGE–TAK1–TAB1 complexes, which in turn promote enhanced NF-κB signalling [107]. A very recent study has greatly expanded the list of potential Fbxo7 substrates. By using a protein array-based screen to identify possible targets of Fbxo7 activity, Teixeira et al. [108] found that Fbxo7 is capable of ubiquitinating over 330 proteins. Importantly, two of the candidates have potential roles in PD, with a kinase that targets α-synuclein (glycogen synthase kinase-3 beta) and a key mitochondrial protein, Tomm20 (Translocase of outer mitochondrial membrane 20) [108].

Fbxo7 is a 522-amino acid, 58.5-kDa protein, belonging to the F-box family. The F-box proteins can be subclassified according to the type of protein–protein interaction domain they harbour. Fbxl proteins contain a leucine-rich repeat; Fbxw proteins have a WD40 repeat, whilst Fbxo proteins have other domains. In the case of Fbxo7, it has an N-terminal Ubl domain, a C-terminal proline-rich repeat, a CDK6-binding region, and an FP domain, in addition to the characteristic F-box (Figure 1). The Fbox is the substrate adaptor that binds to Skp1 to allow integration into the SCF complex. Currently, there are no known mutations within the F-box that are associated with disease. However, there is one homozygous mutation C-terminal to the F-box (R278G) that has reduced binding to Skp1, with no obvious effect on NF-κB signalling [109]. The FP domain is a conserved globular domain [110] found in both Fbxo7 and the Proteasome Inhibitor 31 (PI31) protein. Intriguingly, this domain is a dimerisation domain and blocks proteasome function [111,112]. There are no known pathogenic mutations yet associated with this domain.

The Ubl domain is very distant from ubiquitin, sharing only 22% sequence identity with ubiquitin, and is found at the very N-terminus of Fbxo7. The Ubl domain was initially thought to be important for substrate recruitment, although isoform 2, which lacks the Ubl domain, can still interact with substrates [104,113,114]. Previously, it has been shown that Fbxo7 interacts with Parkin, via the Ubl domain of Fbxo7 [113], and can also interact with PINK1. The interaction between Parkin and Fbxo7 is proposed to facilitate mitophagy by enabling Parkin recruitment to the mitochondria, and overexpression of human Fbxo7 can compensate for the loss of Parkin in a *Drosophila* model. However, PINK1 deficiency is not rescued. The Ubl domain contains one compound heterozygote mutation T22M, which still maintains Parkin interaction [92,113,115], although the overexpressed mutant has decreased stability and altered cellular localisation compared with wild type [115]. Intriguingly, the Ubl domain also houses a second mutation that is potentially protective, Y52C, although the mechanism by which this may be achieved is not yet clear [116]. Finally, the C-terminal proline-rich region (PRR) is required for substrate interaction [100,104]. Interestingly, a pathogenic R498X truncation can no longer recruit Parkin to mitochondria, has lower protein expression, but no clear effect on NF-κB signalling [113,115]. This mutant also displays altered subcellular localisation [115]. The similarities in the behaviour of Ubl and PRR mutants raise the possibility of functional interplay between these domains. Finally, Fbxo7 also contains a region for binding to cyclin-dependent kinase 6 (CDK6) and scaffolds cyclin D/CDK6 assembly, thus regulating the cell cycle [117]. In particular, Fbxo7 plays a role in cell-cycle regulation during erythropoiesis, by binding to and stabilising p27 levels, thus arresting cell-cycle exit [118]. This role appears unrelated to canonical F-box function and serves to demonstrate how much there is to understand about the functions and mechanisms of Fbxo7 in PD and in other contexts of cellular stress.

There are additional polymorphisms found in Fbxo7 that may or may not play a role in disease pathology (comprehensively reviewed in refs [119,120]). A very recent study has provided some long-awaited insights into Fbxo7 function in neurones [121]. Fbxo7-knockout mice display early-onset motor deficits and reduced muscle strength, and die within 4 weeks after birth. Importantly, conditional Fbxo7 knockout in the neurons of older mice also show progressive motor impairment, in sharp contrast with the murine knockout models of Parkin. Intriguingly, Vingill and colleagues have also identified a substrate of Fbxo7's E3 ligase activity, the proteasomal subunit PSMA2, which interacts with the Ubl domain of Fbxo7, thereby providing further physical association between Fbxo7 and the proteasome [121–123]. Finally, loss of Fbxo7 affects the assembly of proteasomes, leading to reduced proteasome activity [121]. This fits well with previous work that shows that proteasomal depletion in mouse neurons leads to neurodegeneration (Bedford et al. [14]).

## Future perspective — technologies and drug discovery

Certainly, the evidence is accumulating that misregulation of the ubiquitin proteasome system is causally linked to Parkinsonian disorders. Whilst α-synuclein misfolding and aggregation is central to the development of sporadic PD, it is still unknown whether ubiquitin pathways controlled by PINK1, Parkin, and Fbxo7 are linked to sporadic disease. Evidence from mice models indicate a genetic interaction between PINK1/Parkin- and α-synuclein-induced neurodegeneration *in vivo*, but the mechanisms are unclear, particularly with regard to mitochondrial substrate ubiquitylation and α-synuclein [124]. Evidence from *Drosophila* studies suggests that convergence may occur at the level of the mitochondrial dynamics [125], whilst mammalian studies indicate that Rab GTPases may be the nexus of PINK1 and α-synuclein signalling [83,126]. Furthermore, a clinico-pathological analysis suggests that p-Ub is increased with ageing in human brains and localised within Lewy bodies [127]. An important next step would be to monitor for Parkin and Fbxo7 activities and altered substrate ubiquitylation in PD patient-derived tissues and cells, and recent technical advances now make this possible. Mass spectrometry ubiquitin technologies using aquapeptides that monitor quantitative changes in substrate ubiquitylation have been particularly showcased for the Parkin pathway and provide a robust unbiased readout of the ubiquitin landscape [128]. More recently, activity-based probes for measuring transthioylation activities of RBR E3 ligases such as Parkin will enable facile assessment of Parkin activity in PD patient tissues and cells [50]. Anti-ubiquityl antibodies have been successfully developed and deployed in the chromatin field to measure histone ubiquitylation [129], and it would be exciting to develop such reagents against Parkin and Fbxo7 substrates as well as against ubiquitylated forms of α-synuclein as potential biomarkers of Parkinson's. Therapeutically there is significant interest in targeting DUBs, and in particular, the development of USP30 inhibitors has moved apace given the primary localisation of USP30 within the mitochondria. USP8 inhibitors may also have therapeutic utility in promoting synuclein endolysosomal degradation and preventing protein aggregation. There has also been some work on activating the pathway with PINK1 activators via a pseudo-substrate approach reported to have therapeutic utility [130]. Similarly, there is much interest in developing small-molecule activators of Parkin, which has been boosted by structural analysis of Parkin. Overall, exploiting genetic advances to better understand the role of ubiquitylation has provided an initial framework of understanding and the fact that ∼40% of young-onset Parkinson's remains genetically unexplained suggests that genetics is likely to further contribute to our understanding of ubiquitin signalling in PD.

## Abbreviations

AIMP2: aminoacyl tRNA synthetase complex interacting multifunctional protein 2
AR-JP: autosomal-recessive juvenile Parkinsonism
CDK6: cyclin-dependent kinase 6
CHIP: C-terminus of Hsc70 interacting protein
cIAP1: cellular inhibitor of apoptosis
CRL: Cullin-RING ligase family
DUBs: deubiquitinating enzymes
ER: endoplasmic reticulum
Fbxo7: F-box only protein 7
FP: Fbxo7/PI31
HECT: homologous to E6-AP carboxy terminus
HURP: hepatoma up-regulated protein
iPS: induced pluripotent stem cell
L-Dopa: L-3,4-dihydroxyphenylalanine
LRRK2: Leucine-rich repeat kinase 2
MDVs: mitochondrial-derived vesicles
MINDY: motif interacting with Ub-containing novel DUB family
MPTP: 1-methyl-4-phenyl-1,2,3,6-tetrahydropyridine
Nedd4: neural precursor cell expressed developmentally down-regulated protein 4
NRAGE: neurotrophin receptor-interacting homologue
Tomm20: translocase of outer mitochondrial membrane 20
PD: Parkinson's disease
PINK1: PTEN-induced kinase 1
PRR: proline-rich region
p-Ub: phospho-ubiquitin
RBR: RING-Between-RING
Ser65: Serine65
SIAH: seven in absentia homolog
TMEM: transmembrane
TRAF2: TNF-α receptor-associated factor 2
UBDs: ubiquitin-binding domains
Ubl: ubiquitin-related domain
UCHs: ubiquitin carboxy-terminal hydrolases
USPs: ubiquitin-specific proteases
